# American and Southern Dental Associations

**Published:** 1888-11

**Authors:** 


(CONTINUED.)

---

### AMERICAN AND SOUTHERN DENTAL ASSOCIATIONS.

JOINT MEETING HELD AT LOUISVILLE, KY., AUGUST 28, 29, 1888.

REPORTED FOR INDEPENDENT PRACTITIONER BY "MRS. M. W. J."

The American and Southern Dental Associations met in joint session at Louisville, Ky., August 28 to September 1, 1888. The Galt House was made general headquarters for the majority of the members of both associations.

The National Association of Dental Faculties and the National Board of Dental Examiners held their sessions in the Galt House parlors. Exhibits, clinics, committee meetings, separate meetings of the two associations, and the joint meetings of both, all found ample accommodations in the elegant building of the Female High School, which had been secured for the occasion.

### ADDRESSES.

Each association having held a meeting at 9 A.M., Tuesday, Aug. 28, they met in joint session at 11 A.M.

The opening proceedings were presided over alternately by Dr. Frank Abbott, President of the American, and Dr. B. H. Catching, President of the Southern Association; Drs. Geo. H. Cushing and L. P. Dotterer, Secretaries, and Drs. J. N. Crouse

(*pro tem.*) and H. A. Lawrence, Treasurers, of the respective associations occupying seats on the platform.

Mayor Jacob and the Rev. Mr. Moore, of Louisville, were escorted to the platform by the executive committee.

The Rev. Mr. Moore delivered a short prayer, asking a blessing upon the work about to be entered upon, after which Mayor Jacob delivered a brief but cordial address of welcome. Dr. A. O. Rawls, on behalf of the Kentucky State Dental Association, gave the associations a most cordial welcome to "hearts sincere and hands outstretched," to which Dr. E. G. Darby, of the American, and Prof. J. Y. Crawford, of the Southern, responded.

Dr. Darby, in accepting the hospitality of Kentucky, said that it had been observed that if a distinguished gentleman was in search of a beautiful wife, to shine at the capital of the nation, he went to Kentucky for her. If a millionaire politician wishes to present the Chief Executive with a pair of thoroughbred horses, he goes to Kentucky for them. If an epicurean tippler wishes to tickle his own palate, he sends to Kentucky for the wherewithal.

Prof. Crawford dwelt at some length upon the illustrious names Kentucky has furnished to the rolls of medicine, surgery, and dentistry.

At the conclusion of his address the meeting adjourned to 7.30 P.M., when the two Presidents delivered their addresses.

Dr. Frank Abbott, President of the American Association, called attention to the advances made by the profession in the past few years, the increased facilities for a thorough dental education, brilliant achievements of American dentists, and the giant strides made through microscopical researches.

Dr. B. H. Catching, President of the Southern Association, took for his topic the question: "Is the average dentist of to-day a specialist in medicine?" He took the ground that the present system of dental college education does not raise the student to a sufficiently elevated scientific standing. The average dentist looks upon his vocation as a mechanical trade rather than a scientific specialty. While mechanical ability is requisite, a scientific knowledge of medicine and surgery is essential. The average dentist is proficient in that which equips the artisan; but deficient in that which entitles him to recognition as a specialist in medicine. He considers the present system of dental education too narrow and contracted to permit the highest conceptions of the possibilities of our grand calling. Higher qualifications, a broader curriculum, an extension of time, are the demands of to-day. The surgical part of dentistry is its most distinctive feature. The general surgeon is master of medical science in so far as it is taught in the medical schools, and so of all specialists in medicine—the occulist, the otologist, the dermatologist, and all the others—the dental surgeon alone is not master of medical science, because he is not taught in the medical school. Hence he reaches the conclusion that the dental student should first pass through the medical college and receive the degree of M.D., and then enter the dental infirmary, where he would be taught the practical methods of the dental surgeon, the manipulative art of the dental operator. He also spoke of the importance of a more thorough training in medical science in view of legal complications, the degree of M.D. affording protection in certain contingencies which the degree of D.D.S. alone does not secure.

This paper elicited a very animated discussion, participated in by Profs. Truman, Morgan, Crawford, Pierce, Carpenter, Drs. N. J. Roberts, Cushing, J. J. R. Patrick, Brophy, H. A. Smith, Ottofy, Jas. Johnson, Sudduth, Freeman, Friedrichs, Peabody and others. While it was universally admitted that a certain degree of medical science and knowledge of Materia Medica was necessary, it was held that this was included in the curriculum of the dental college. To the dentist mechanical skill is essential, and a training of hand and eye, which is not to be looked for in a medical school. Dr. Roberts thought that the average dentist, who had not been educated in general medicine, would not be able to follow out the line of treatment in cases of necrosis, blood-poisoning, tetanus, etc., and without the degree of M.D. would have no protection in case of fatal results.

Dr. J. J. R. Patrick said that neither common law nor criminal law would hold a man guiltless, if guilty, no matter how many diplomas he might hold, or how many degrees he might claim.

Dr. Brophy said that a man who had been educated for a certain line had a right to follow that line. Under the diploma of a certain institution he had a right to practice anything taught in that institution.

Dr. Sudduth said that everything depended on the ability of the individual. If the operation performed was not taught in the college from which he held his degree, the jury would be prejudiced by that fact; but if he could show that he was skilled and competent, the prejudice could be overcome.

In closing the discussion, in which many other points were brought out, Dr. Catching said that one fact was patent. If a physician administers chloroform with fatal results, he has only to say: "Heart failure!" and he is all right. On the other hand, if the dentist meets with the same misfortune, the physician has to give the certificate, and if it is adverse to the D.D.S. he goes behind the iron bars. Dr. Catching humorously congratulated his opponents on the fact that the majority of them were college professors, with the degree of M.D., to which many of then attached so much importance that they ignored their D.D.S. entirely.

JOINT MEETINGS FOR PAPERS AND DISCUSSIONS.

Joint meetings of both Associations were held daily at 9.30 A. M. and 7.30 P. M., to hear the reports of standing committees, reading of papers recommended by the committee, and discussion of the same.

All of the papers presented by a committee were read in succession, the discussion covering all of the subjects brought forward in the different papers. This plan, though open to some objections, was adopted as the only practicable method of getting through with the vast amount of work before the two Associations. Each committee was represented by a chairman from both Associations.

EVENING SESSION, AUGUST 28.

The report of the Committee on Operative Dentistry was called for.

GEO. H. WINKLER, *South. Asso.*, }

E. T. DARBY, *Amer. Asso.*, } Chairmen.

*Dr. Darby* responded in the absence of Dr. Winkler, to whom it had been allotted.

*Dr. Darby* reported four papers and some appliances. The subjects attracting most attention were immediate-root-filling, implantation and the use of copper amalgam. The great advances made in the use of germicides has so removed the danger of septic contagion, that great operations are now safely undertaken. Implantation is undoubtedly a success for the time being; but time alone can solve the problem as to its durability. Copper amalgam has made its way, and retains its position as the best filling material in a certain class of cases.

*Dr. W. Storer How* read a paper on

PORCELAIN INLAYS, illustrated by numerous specimens, which were handed around for inspection. The details for manipulation, both of cavity and of porcelain stopper, are substantially the same as given in the *Cosmos*, July issue.

*Dr. J. J. R. Patrick* read a paper on

METHODS OF MOUNTING ARTIFICIAL CROWNS, the method being practically as reported in the proceedings of the Illinois State Society in its recent meeting at Cairo.

*Dr. C. E. Kells, Jr.*, made a number of interesting experiments, illustrating

THE CONDUCTIVITY OF FILLING MATERIALS, by means of a very delicate thermostat, the expansion of the different materials ringing an electric bell at varying intervals of time, according to the conductivity of the material.

*Dr. H. Gilmer* read a paper describing his method of making cheap crowns, which are a recognized necessity for many of our patients who cannot afford the more costly gold and porcelain crowns. In Dr. Gilmer's method a platinum band is fitted to the prepared root, and filled with modeling compound, the patient biting to give the correct articulation. This is removed from the mouth, and, after any necessary trimming, invested in plaster. This is drilled, the wax removed, and Weston's or Watt's metal poured, making a low-priced, easily made, serviceable crown for the posterior teeth. For the anterior teeth a low carat gold band may be used, with porcelain facing. The subject of crowns and the preparation of roots, called forth a lengthy discussion of the proper methods of dealing with exposed pulps—whether too many pulps were not being ruthlessly destroyed to make place for the pins and posts of artificial crowns, and whether it is not time to call a halt and return to more conservative methods. The discussion showed that the younger members as a rule repudiate pulp capping *in toto*, while the older members advocate the preservation of the pulps.

*Drs. C. N. Pierce*, *W. H. Atkinson*, *Allport*, *Freeman*, *Morgan*, *Taft* and *E. T. Darby* spoke eloquently and earnestly in favor of giving the pulp every possible chance.

*Dr Pierce* would cauterize the pulp, and so get a non-conducting surface under the filling.

*Dr. Atkinson* touches the pulp with creosote and places over it a concave cap of gold.

*Dr. Freeman* does not wish to kill a pulp with arsenic. If they are going to die they will die more kindly, the kinder we treat them.

*Dr. Taft* considers that devitalization is followed be deterioration. There is loss of continuity of structure; the tooth breaks down, independent of decay; it wears rapidly under attrition, and seems to wear away. The pulp furnishes the nutrient material through which the dentine lives and retains its sensitiveness.

*Dr. Morgan* thinks that, though the cememtum continues to be nourished by the pericementum after the death of the pulp, so that a pulpless tooth is not necessarily "a dead tooth;" it is nevertheless partially devitalized, and consequently not in as good condition as one which is alive in all its parts.

*Dr. Patrick*, on the other hand, considers it contrary to all experience, contrary to physiology, that a ruptured, bleeding pulp can recover; that it has never been proved that it is possible. The office of the pulp is to produce dentine, and after that has been accomplished all the pulp can do is to wall itself in, dentinification continuing from the circumference clear to the centre; the roots in old people being sometimes completely filled up.

*Dr. Cravens* said that from the time the tooth is formed the pulp labors to kill itself, by progressive calcification, the dentine becoming quite insensible and the tubuli all plugged up from receiving no nutrition. He is convinced that in the mature tooth the pulp is of little essential service.

*Dr. Kills* said that in a hundred cases of pulp-capping he would look for ninety-nine failures.

*Dr. Story* (Texas) has never been able to save a diseased pulp. He uses arsenic every time and fills with oxychloride of zinc, and never has any further trouble.

*Dr. Darby* has been very successful for twenty-five years. He applies to the surface of the pulp a soft paste of oxide of zinc and creosote, followed by oxychloride, finishing usually with gold. He has one case in which a pulp has been capped three times, from different cavities of decay, and is living now, responding to every test.

*Dr Truman* had not found it possible to save seventy-five per cent. of exposed pulps. If the pulp has been irritated and congested he deems it next to impossible to save it. Where the individual possesses extreme vitality and all conditions are favorable, it may be possible to save an exposed pulp; but it is not the rule. He does not agree with Dr. Taft as to the deterioration of pulpless teeth. The dentine is covered by the cememtum, which is nourished by the pericementum, and so kept in health.

HISTOLOGY AND MICROSCOPY.

*Dr. Wilson,* Burlington, Iowa, read a short paper on The Apical Portion of the Cementum in its Physiological and Pathological Relations.

After describing the structure of cementum, and the function of its surrounding membrane, he said that the main point of interest was the fact that though a devitalized pulp does not interfere with the vitality of the cementum, yet in destroying the pulp the apical portion of the cementum is sometimes destroyed, and necrosis ensues, through the effect of arsenic applied to the pulp, reaching the cementum through the canaliculi. A portion of the cementum being dead, nature must dispose of it; this is accomplished through solution of the lime salts. For this reason arsenic should be used with great caution. If the first application fails there is danger in a second, and more so in a third application. Many incurable abscesses are the result of arsenical poisoning.

*Dr. Atkinson* said this paper had a close bearing on the occult movements in nutrition and de-nutrition.

We may burr out all diseased tissues in pulpless teeth—one-eighth or one-quarter of an inch without fear; then disinfect and leave.

Arsenic acts through the affinity of arsenious acid for the protoplasmic tissue in the pulp, and its activity depends upon how much semi-fluid material there is. If only the legitimate quantum it will sleep till Gabriel's trump.

How many have extracted a tooth, felt of the end of the root, and finding it rough, say: "Yes, that tooth had to come out." But if he had got at the end of the root and treated it properly, it would have been all right in its own place.

*Dr. Frank Abbott*, read a paper entitled "The Odontoblasts in Relation to Developing Dentine."

Dr. Abbott first reviewed the secretion theory, to which he, with many others held, for a long time, but which had never satisfied him, and which he had abandoned for the transformation theory, especially as applied to developing dentine, which surpasses in refinement of structure all other.

The main mass is not cartilaginous, but glue-yielding, infiltrated with lime salts. The only cells are the odontoblasts, which send offshoots upward and downward to the papilla, only the outer layer of the pulp only being concerned in forming dentine. He denies that the whole pulp is transformed.

Dr. Abbott quoted the theories of Drs. Andrews, Bodecker and Heitzman, as to the origin of the dental fibrile, whether they are the remnants of the central portions of the odontoblasts or have their origin between the latter, Andrews maintaining that only certain pear-shaped odontoblasts are fibril cells. Bodecker and Heitzman that the odontoblasts split up into medullary or embryonal tissue corpuscles, the living matter remaining unaltered. (See article on eburnitis.) The return of the odontoblasts to mudullary tissue, before infiltration with lime salts, solves the mooted question as to the continuous mass of dentine structure, it being only exceptionally stratified, as in a cusp.

The discussion which followed the reading of this paper was confined to Drs. Sudduth and Abbott, the former maintaining the secretion theory, especially for the hard tissues, through the deposition of lime salts, the action of the odontoblasts in the dentine, of the ameloblasts in the enamel, of the osteoblasts in bone. He said that Dr. Abbott made no distinction between the cortical and cancellous portions of bone, these being secreted in different ways. In the cortical portions a sheath is laid down, from a membranous, in such a way as to forn a cortically layer which is secreted with regularity, and only in certain lines and certain places ; and so of dentine—there is a power which directs this secretion, which does not occur indiscriminately. He said that Dr. Abbott also ignored the nuclear structure of tissues. Dr. Sudduth said that he had visited the laboratories of Drs. Bodecker and Hirtzman, and been shown some of their specimens; but was unable to see whence they drew their conclusions. In specimens carefully stained the nucleus can be clearly seen, but in their specimen it could not be made out, so that they proved nothing The study of the secretion of shells substantiates the theory of the secretion of enamel and other hard tissues.

Dr. Abbott, in reply, said that if we did not differ in opinion, we should never learn anything. Different men can look at the same thing, with the same glass, and yet see and understand in widely different ways. No two will see alike. The secretion theory contemplates a row of odontoblasts, their distal ends in contact with the ameloblasts, and which secrete lime salts and all other substances which fall back and are found around the pulp. Kollicker concluded, in 1850, that this could not be ; that the odontoblast itself becomes dentine, another row forming underneath, the nucleus splitting up again and again and the odontoblasts increasing in length. This seems more rational than the secretion theory. Another theory is that the odontoblasts become calcified, beginning at the centre and working to the periphery ; this is denied by many. In every odontoblast there may be discovered a reticulum full of little nuclei ; but this is only a provisional step in the formation of dentine.

There is a layer of tissue between that inhabited by organisms and perfect dentine, that has never been explained except on the reaction theory. In the process of caries, if we take a section of dentine with the cavity in it, we find the first layer of carious substance soft, and filled with micro-organisms—micrococci and leptothrix principally—deeper in we find a layer, which by process of staining colors different from the other—micro-organisms penetrate to a certain extent—but as we go further we find a yellow stain; the line of demarcation is very plain. We find dentine broken down, disorganized, yet not an organism.

The living matter—glue-giving basis substance—responds to irritation, takes on inflammatory conditions, but organisms do not cause decay; they come there because there is decay.

*Dr. Sudduth* said that no theory ever yet advanced explained the production of the cavity, except the germ theory of Dr. Miller of Berlin. Lactic acid dissolves lime salts, but the form of the dentine is unchanged. Certain bacteria of the human mouth, developing in decalcified dentine throw out a digestive ferment, which breaks down the form of the tooth. The age of decay gives the different colors—yellow first—as seen by Dr. Abbott. Dr. Miller stands pre-eminent in this line of investigation and discovery, and as time rolls on he will get the credit due him.

*Dr. Abbott* replied that the lime salts are not dissolved out; we find under the excavator that they are there, though broken down. In the cavity everything is gone. Whether micro-organisms have chewed it, or whether they are at all to blame for all this, is of no importance. If the teeth are kept perfectly clean they will not decay; there are undoubtedly organisms in the mouth as everywhere else.

*Dr. Sudduth.*—If you do not give credit to the part micro-organisms play in decay, then how do you explain the formation of the cavity?

*Dr. Abbott.*—No cavity is formed except by acids ; but whether from decomposing food or from micro-organisms is indifferent to me. When it gets through the enamel it reaches tissue composed of living matter; this becomes irritated; by the inflammatory process lime salts are displaced, melted down, and embryonal tissue is formed; the dissolved lime salts are washed out and carried away the same as any other product.

*Dr. Sudduth.*—The admission that the breaking down may be due to micro-organisms is the very thing claimed. A tooth may remain indefinitely in putrifying mixtures and no effect will be had on it. There is only one form of micro-organism that can produce the dissolution of the basis substance and form a cavity.

*Dr. Abbott*, in conclusion, denied that he had made the admission accredited him by Dr. Sudduth.

[TO BE CONTINUED.]

